# Fast tracking in cardiac surgery: is it safe?

**DOI:** 10.1186/s13019-022-01815-9

**Published:** 2022-04-06

**Authors:** Jeffrey B. MacLeod, Kenneth D’Souza, Christie Aguiar, Craig D. Brown, Zlatko Pozeg, Christopher White, Rakesh C. Arora, Jean-François Légaré, Ansar Hassan

**Affiliations:** 1 Cardiovascular Research New Brunswick, Saint John Regional Hospital, Saint John, NB Canada; 2 Cardiovascular Research New Brunswick, and Department of Cardiac Surgery, Saint John Regional Hospital, Saint John, NB Canada; 3 Max Rady College of Medicine, Department of Surgery, University of Manitoba, St. Boniface Hospital, Winnipeg, MB Canada; 4 Department of Cardiovascular Surgery, Maine Medical Center, Portland, Maine USA

**Keywords:** Outcomes, Patient safety, Postoperative care

## Abstract

**Background:**

While fast track clinical pathways have been demonstrated to reduce resource utilization in patients undergoing cardiac surgery, it remains unclear as to whether they adversely affect post-operative outcomes. The purpose of this study was to determine the impact of fast tracking on post-operative outcomes following cardiac surgery.

**Methods:**

In a retrospective study, all patients undergoing first-time, on-pump, non-emergent coronary artery bypass grafting, valve, or coronary artery bypass grafting + valve at a single centre between 2010 and 2017 were included. Patients were considered to have been fast tracked if they were extubated and transferred from intensive care to a step-down unit on the same day as their procedure. The risk-adjusted effect of fast tracking on a 30-day composite of all-cause mortality, stroke, renal failure, infection, atrial fibrillation, and readmission to hospital was determined. Furthermore, propensity score matching was used to match fasting track patients in a 1-to-1 manner with their nearest “neighbor” in the control group and subsequently compared in terms of 30-day post-operative outcomes.

**Results:**

3252 patients formed the final study population (fast track: n = 245; control: n = 3007). Patients who were fast tracked experienced reduced time to initial extubation (4.3 vs. 5.6 h, p < 0.0001) and lower median initial intensive care unit length of stay (7.8 vs. 20.4 h, p < 0.0001). Fast tracked patients experienced lower 30-day rates of the composite outcome (42.4% vs. 51.5%, p = 0.008). However, following propensity score matching, fast tracked patients experienced similar 30-day rates of the composite outcome as the control group (42.4% vs. 44.5%, p = 0.72). After risk adjustment using multivariable regression modeling, fast tracking was predictive of an improved 30-day composite outcome (OR 0.75, 95% CI 0.57–0.98, p = 0.03).

**Conclusion:**

Fast track clinical pathways was associated with reduced intensive care unit, overall length of stay and similar 30-day post-operative outcomes. These results suggest that fast tracking appropriate patients may reduce resource utilization, while maintaining patient safety.

**Graphical abstract:**

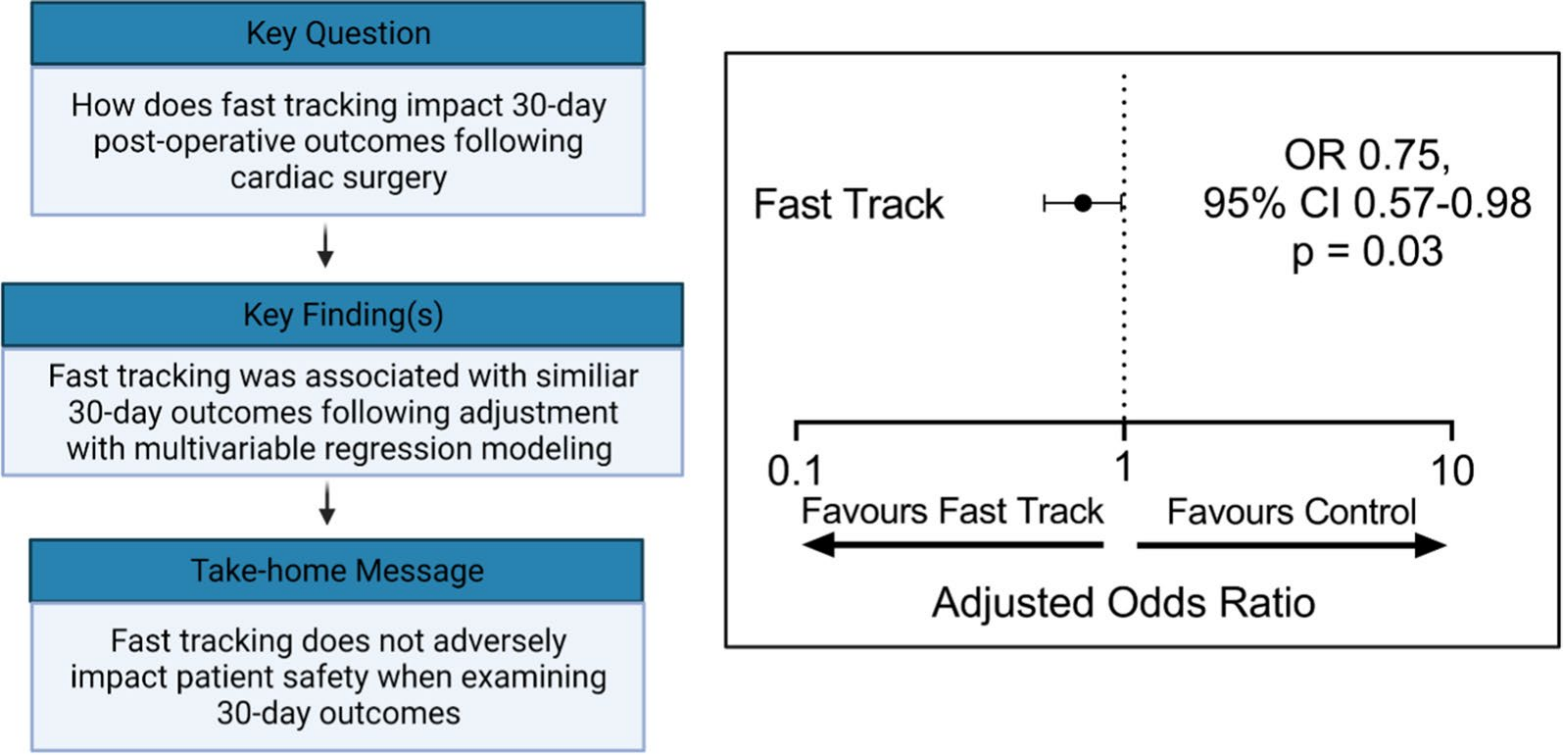

**Supplementary Information:**

The online version contains supplementary material available at 10.1186/s13019-022-01815-9.

## Background

Historically, patients undergoing cardiac surgery were administered high-dose, long-lasting, opioid-based anaesthesia and post-operative analgesia [1]. While helping to address the complex needs of early post-operative care of patients, this regimen also contributed to patients being mechanically ventilated for extended periods of time in the intensive care unit (ICU) before finally being extubated and transferred to the ward 24–48 h following their surgery. Unfortunately, this approach, combined with a rise in patient complexity and an overall increase in the number of patients undergoing cardiac surgery, has led to a greater strain on the health care system.

To alleviate this burden, clinical pathways to fast-track patients following cardiac surgery were introduced, whereby patients were given low-dose or short-acting anesthetic agents, extubated early (i.e. less than 6 h following their operation) and then transferred from the ICU to the step-down or intermediate-care unit on the same day as their surgery [2–6]. The primary goal of this “day zero” fast track pathway was to reduce ICU and hospital length of stay, therein minimizing resource utilization and improving overall efficiency of patient recovery [6–8].

While fast track pathways have been shown to be associated with reduced time to extubation and decreased initial ICU length of stay (LOS) [9–11], the uptake of fast track has been variable. This inconsistency has been attributed to differences in practice patterns, lack of availability of step-down or intermediate-care resources and concerns regarding safety due to differing levels of monitoring and nursing-to-patient ratios in the ICU as compared to the intermediary or step-down units. Therefore, the purpose of this study was to determine the impact of fast-track on 30-day post-operative patient and hospital outcomes following cardiac surgery.

## Methods

This was a retrospective, single center study.

### Patients

**Fig. 1:**
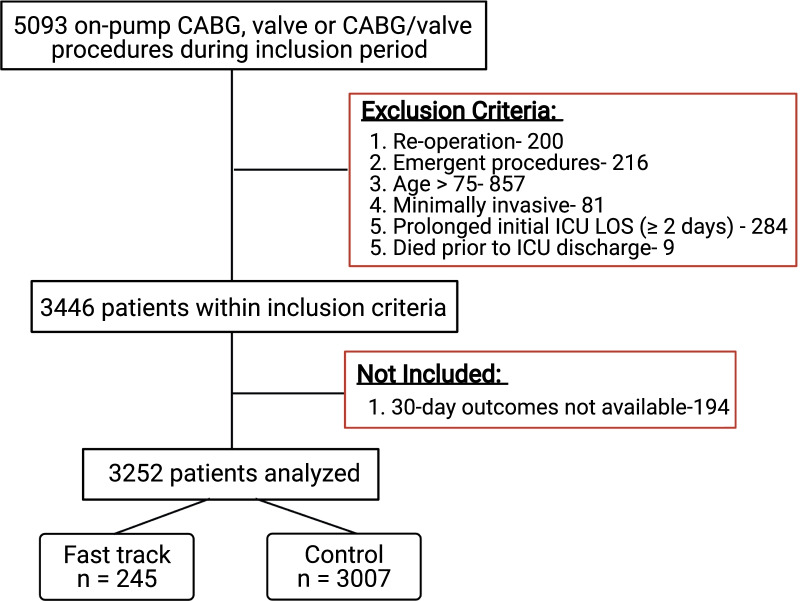
Inclusion/exclusion criteria

All patients having undergone first time, non-emergent, on-pump coronary artery bypass grafting (CABG), valve, or combined CABG/valve surgery between January 1, 2010 and December 31, 2017 at the New Brunswick Heart Centre (NBHC) in Saint John, New Brunswick were retrospectively considered for inclusion. Patients were excluded if they were over the age of 75, underwent minimally invasive surgery, experienced a prolonged initial ICU LOS ≥ 2 days or died prior to discharge from the ICU. These patient groups were excluded to avoid confounding our results with potentially highly variable clinical courses. Patients were also excluded if they did not have complete 30-day follow-up. Based on our inclusion/exclusion criteria, 3252 patients formed the final study population (Fig. 1). The study was undertaken as a part of an internal quality improvement project within the department. Due to the retrospective and database driven nature of the project, we did not seek REB approval.

### Variables

Following assessment of the above-mentioned inclusion and exclusion criteria, patients who formed the final study population were divided into two groups: those who were successfully fast tracked following their cardiac surgery (fast track group) and those who were not (control group). Groups were compared in terms of baseline and intraoperative characteristics as well as in-hospital and 30-day post-discharge outcomes. All data were obtained from the NBHC Cardiac Surgery Registry, a detailed observational registry that includes all cardiac surgery encounters at the NBHC.

### Outcomes

The primary outcome of interest was a 30-day composite of mortality, infection (sternum and leg incision sites, UTI, or sepsis), atrial fibrillation, renal failure, stroke (permanent or transient), and readmission to hospital. Infection was defined as the presence of positive cultures or treatment with antibiotics. Renal failure was identified in patients with a post-operative serum creatinine of > 176 µmol/L or 40 µmol/L greater than baseline in patients with pre-existing renal failure. Readmission was defined as an in-patient visit to any hospital for any reason following the date of initial discharge from hospital and within 30 days from the date of surgery.

### Fast tracking

No major changes to our fast-track policy occurred during the study period. All patients undergoing cardiac surgery were considered for fast tracking upon their admission to the ICU at the NBHC, an academic, teaching hospital that is the sole provider of tertiary cardiac care services for the province of New Brunswick, in Canada. The ICU at the NBHC is an open-model ICU where the attending surgeon serves as the most responsible physician throughout the patient’s ICU stay. The ICU is not staffed with residents, fellows, nurse practitioners or physician assistants, and the decision as to whether or not to attempt to fast track is made by the attending surgeon alone. During the period of study, there was no formal protocol to guide the fast track decision-making process; however, the decision to activate the fast track pathway was made in a patient-tailored manner that was based on case complexity, hemodynamic stability and degree of post-operative bleeding as well as individual practitioner preference, rather than hospital policy. The ability to fast track a patient was further dependent on the availability of a bed in the step-down unit. If a bed was not available, then the patient was kept in the ICU until the next day despite being ready appropriate for fast tracking.

Patients were considered to have been successfully fast tracked if they were extubated and transferred to the step-down unit on the same day as their procedure (i.e. a day zero discharge from the ICU). It should be noted that the step-down unit at the NBHC has a nurse-to-patient ratio of 1:2 (as opposed to 1:1 in the ICU) and is capable of monitoring arterial blood pressure and central venous pressure monitoring lines and accepting patients on limited inotropic and/or vasopressor support. Furthermore, the attending surgeon continues to function as the most responsible physician while the patient is in the step-down unit.

### Statistical methods

Categorical variables were summarized as numbers of observations and percentages. Continuous variables, on the other hand, were summarized as means and standard deviations for normally distributed data and as medians and interquartile ranges for non-normal data. Chi square and Fisher’s exact tests were used to assess group differences in categorical variables, while Welch’s t-tests and Kruskal–Wallis tests were used for continuous variables. P-values < 0.05 was considered statistically significant.

The impact of fast tracking on the composite outcome was determined using multivariable regression modeling, first including all baseline and intraoperative characteristics as potential confounders and then using backward elimination to refine the model based on Akaike information criterion. Goodness-of-fit was demonstrated using the concordance (C) statistic.

Propensity score matching was also used to isolate the effect of fast tracking. Propensity scores were estimated for each patient using multivariable logistic regression modeling on the basis of baseline and intra-operative characteristics. Using these scores, patients in the fast track group were then matched in a 1-to-1 manner with their nearest “neighbor” in the control group and subsequently compared in terms of 30-day post-operative outcomes.

All analyses were performed using R version 3.6.2 (R Core Foundation, Vienna, Austria).

## Results

During the study period, 3252 patients formed the final study population (fast track: n = 245; control: n = 3007). Baseline characteristics are shown in Table 1. Overall, no statistically significant differences between fast track and control patients were observed in terms of age, sex, or distribution for most risk factors/comorbidities, including smoking history, hypertension, dyslipidemia, diabetes and renal failure. Fast tracked patients did however have lower rates of baseline cerebrovascular disease, chronic obstructive pulmonary disease (COPD) and an EF < 40% but higher rates of unstable angina and NYHA-IV symptoms.

**Table 1:** Baseline characteristics

Characteristics, n (%)	Fast track (n = 245)	Control (n = 3007)	p value
Age, years, mean ± SD	63 ± 8	63 ± 8	0.27
Age ≥ 70 years	58 (23.7)	724 (24.1)	0.95
Female sex	41 (16.7)	558 (18.6)	0.53
Smoking history	165 (67.3)	1996 (66.4)	0.81
Hypertension	174 (71.0)	2141 (71.2)	1.00
Dyslipidemia	189 (77.1)	2340 (77.8)	0.87
Diabetes	88 (35.9)	1076 (35.8)	1.00
CVD	15 (6.1)	318 (10.6)	0.04
PVD	19 (7.8)	340 (11.3)	0.11
Recent MI ≤ 21 days	84 (34.3)	946 (31.5)	0.40
Renal failure	2 (0.8)	79 (2.6)	0.12
Unstable angina	137 (55.9)	1426 (47.4)	0.01
Atrial fibrillation	12 (4.9)	210 (7.0)	0.27
COPD	11 (4.5)	297 (9.9)	0.008
NYHA class 4	167 (68.2)	1706 (56.7)	0.0006
EF < 40%	10 (4.1)	303 (10.1)	0.003
Urgent status	161 (65.7)	1631 (54.2)	0.0007

COPD, chronic obstructive pulmonary disease; CVD, cardiovascular disease; EF, ejection fraction; MI, myocardial infraction; NYHA, New York Heart Association; PVD, peripheral vascular disease

When examining intraoperative characteristics (Table 2), fast tracked patients were more likely to have undergone isolated CABG and less likely to have undergone isolated valve or CABG + valve surgery. Cardiopulmonary bypass (CPB) and cross clamp (XC) times were significantly lower in fast track vs. control patients. Fast tracked patients were also less likely to require the use of inotropes upon transfer to the ICU.

**Table 2:** Intraoperative characteristics

Characteristics, n (%)	Fast track (n = 245)	Control (n = 3007)	p value
*Procedure*			
CABG	220 (89.8)	2375 (79.0)	< 0.0001
Valve	21 (8.6)	364 (12.1)	
CABG + valve	4 (1.6)	268 (8.9)	
CPB time, mdn (IQR)	79 (64–95)	92 (74–113)	< 0.0001
XC time, mdn (IQR)	60 (46–73)	69 (52–88)	< 0.0001
Inotropes	29 (11.8)	546 (18.2)	0.02

CABG, coronary artery bypass graft; CPB, cardiopulmonary bypass; XC- cross clamp

In-hospital outcomes are presented in Table 3. Fast tracked patients were extubated significantly earlier than control patients and experienced shorter initial ICU LOS. Additionally, fast tracking was significantly associated with shorter postoperative LOS and the decreased likelihood of postoperative LOS > 5 days. While fast track was associated with lower rates of ICU readmissions, this difference did not reach statistical significance.

**Table 3:** In-hospital outcomes

Characteristics, n (%)	Fast track (n = 245)	Control (n = 3007)	p value
*Adverse post-operative outcome*			
Mortality	0 (0.0)	23 (0.8)	0.41
Reop for bleeding	0 (0.0)	17 (0.6)	0.63
Wound infection	13 (5.3)	88 (2.9)	0.06
UTI	1 (0.4)	23 (0.8)	1.00
Sepsis	0 (0.0)	9 (0.3)	1.00
Atrial fibrillation	70 (28.6)	1119 (37.2)	0.008
Renal failure	6 (2.4)	152 (5.1)	0.09
CVA	1 (0.4)	17 (0.6)	1.00
TIA	0 (0.0)	11 (0.4)	1.00
*ICU resource utilization*			
Initial vent time, h	4.3 (3.3–5.2)	5.6 (4.4–7.7)	< 0.0001
Reintubation	0 (0.0)	5 (0.2)	1.00
BiPAP	0 (0.0)	1 (0.0)	1.00
Initial ICU time, h	7.8 (6.5–9.4)	20.4 (17.3–22.1)	< 0.0001
Prolonged ventilation > 24 h	0 (0.0)	8 (0.3)	1.00
Readmission to ICU	0 (0.0)	40 (1.3)	0.07
*Hospital resource utilization*			
Postop LOS, days, median (IQR)	5 (4–6)	5 (4–6)	0.0001
Postop LOS > 5 days	72 (29.4)	1205 (40.1)	0.001
*Discharge disposition*			
Expired	0 (0.0)	23 (0.8)	0.0007
Home	212 (86.5)	2344 (78.6)	
Home EMH	12 (4.9)	279 (9.3)	
Other service	2 (0.8)	12 (0.4)	
Other institution	19 (7.8)	349 (11.7)	

BiPAP, bilevel Positive Airway Pressure; CVA, cerebrovascular accident; EMH, extramural home care; TIA, transient ischemic attack; UTI, urinary tract infection

Unadjusted 30-day rates of the composite outcome were significantly lower among fast tracked patients as compared to control patients, a finding that was driven in large part by the lower rates of readmission and atrial fibrillation among fast tracked patients (Table 4). Following adjustment for differences in baseline characteristics, fast track was independently associated with improved 30-day rates of the composite outcome of interest (Table 5).

**Table 4:** 30-day postoperative outcomes

Characteristics, n (%)	Fast track n = 245	Control n = 3007	p value
Composite^a^	104 (42.4)	1549 (51.5)	0.008
Mortality	0 (0.0)	26 (0.9)	0.26
Wound infection	29 (11.8)	353 (11.7)	1.000
UTI	4 (1.6)	36 (1.2)	0.54
Sepsis	0 (0.0)	9 (0.3)	1.00
Atrial fibrillation	70 (28.6)	1170 (38.9)	0.002
Renal failure	6 (2.4)	156 (5.2)	0.08
CVA	3 (1.2)	32 (1.1)	0.74
TIA	1 (0.4)	21 (0.7)	1.00
Readmission	13 (5.3)	308 (10.2)	0.02

^a^Mortality, wound infection, UTI, sepsis, atrial fibrillation, renal failure, CVA, TIA, readmission

**Table 5:** Logistic regression (30-day composite)

Variable	OR	95% CI		p value
		2.5	97.5	
Fast track	0.75	0.57	0.98	0.03
Age, years	1.05	1.04	1.06	< 0.0001
Hypertension	1.26	1.07	1.48	0.005
CVD/PVD	1.33	1.10	1.60	0.004
COPD	1.31	1.02	1.68	0.03
NYHA class 4	1.25	1.08	1.45	0.003
*Procedure*				
CABG	1.00	-	-	-
Valve	1.74	1.38	2.20	< 0.0001
CABG + valve	1.65	1.26	2.16	0.0003

Other variables considered: female sex, smoking history, dyslipidemia, diabetes, MI <  = 21 days, renal failure, EF < 40%, urgent status, inotropes after OR. C = 0.64

Propensity score matching was used to create 245 matched pairs of patients that were fast tracked and those that were not. Baseline characteristics, intraoperative characteristics and post-operative outcomes were similar in matched patients, with the exception of initial time to intubation, initial ICU LOS and postoperative LOS, all of which were significantly lower among fast tracked patients (Additional file 1: Tables S1–S3). Re-examination of the 30-day composite of post-operative outcomes demonstrated no difference between fast tracked patients and their matched controls (Table 6).

**Table 6:** 30-day postoperative outcomes, matched

Characteristics, n (%)	Fast track n = 245	Control n = 245	p value
Composite^a^	104 (42.4)	109 (44.5)	0.72
Mortality	0 (0.0)	1 (0.4)	1.00
Wound infection	29 (11.8)	26 (10.6)	0.77
UTI	4 (1.6)	5 (2.0)	1.00
Sepsis	0 (0.0)	0 (0.0)	1.00
Atrial fibrillation	70 (28.6)	82 (33.5)	0.28
Renal failure	6 (2.4)	5 (2.0)	1.00
CVA	3 (1.2)	2 (0.8)	1.00
TIA	1 (0.4)	0 (0.0)	1.00
Readmission	13 (5.3)	22 (9.0)	0.16

^a^Mortality, wound infection, UTI, sepsis, atrial fibrillation, renal failure, CVA, TIA, readmission

## Discussion

Fast tracking in cardiac surgery was introduced as a mechanism by which patients could be extubated early and transferred out of the ICU on the day of their procedure. By reducing ICU and potentially hospital LOS, it was anticipated that fast tracking would reduce resource utilization and improve overall hospital efficiency. However, the lack of consensus regarding the safety of fast tracking as it related to post-operative outcomes has led, in part, to the inconsistent uptake of this strategy. In this study, we demonstrated that fast tracking was associated with significant reductions in intubation time, initial ICU LOS and post-operative LOS, and following risk-adjustment was associated with similar 30-day post-operative outcomes.

The association between fast tracking and reduced ICU resource utilization, as shown in this study, has been previously seen [11–14]. While the reductions in initial ICU LOS were largely driven by differences in initial ventilation times in those studies [11, 13, 15, 16], this is not what we observed. Rather, we found that differences in initial ventilation times between fast track patients and the control patients were not as pronounced, especially when compared to differences in initial ICU LOS. The overall extubation times in both groups reflect the aggressive approach taken by our institution to extubate patients early regardless of whether they are to be fast-tracked or not. The question that arises though is why control patients were not fast-tracked despite their having been extubated relatively early. It may be postulated that they were not fast-tracked due to a variety of non-clinical reasons including bed unavailability, the time of day at which these patients were extubated (i.e. patients were less likely to be fast tracked if they were extubated in the evening as opposed to earlier in the day) and practitioner unwillingness to fast track certain patients despite their being eligible.

The observation in this study that fast tracking reduced overall hospital LOS contrasts the findings of a meta-analysis of 28 studies (n = 4438) that showed no association between fast tracking and overall hospital LOS [11]. Multiple factors may negatively affect the ability of a fast-tracked patient to be discharged home earlier as compared to non-fast-tracked patients. For example, the rates of fast track “failures”, where a patient requires re-intubation or re-admission to the ICU, varies between 3.1 and 14.6% [17–21]. In these cases, fast track failure would not only increase ICU resource utilization, but it would also likely necessitate an increase in overall hospital LOS. In our study, no fast-tracked patients required reintubation or readmission to the ICU. As such, the low rate of fast-track failure in our study may have contributed to fast track patients experiencing a shorter overall hospital LOS.

The risk-adjusted effects seen in 30-day rates of the composite outcome of interest supports the notion that fast tracking is indeed safe and does not adversely impact patient safety. Overall, this corroborates findings from previous studies that demonstrated that fast tracking, using either low dose opioid use or time-directed extubation, is not associated with differences in major post-operative complications including stroke (transient or permanent), renal failure, wound infection or hospital readmission [11, 22–24]. We further demonstrated that rates of atrial fibrillation, the most common post-operative complication seen in patients undergoing cardiac surgery, was not affected by fast tracking. To the best of our knowledge, this is the first study to examine the impact of fast tracking on rates of atrial fibrillation. Ultimately, the independent association of fast tracking and rates of post-operative adverse events as seen in this study is of great relevance given that concerns regarding fast tracking and patient safety have likely led to the inconsistent and somewhat limited uptake of fast tracking by many surgeons.

This study is not without its limitations. First, the lack of a formal protocol for fast tracking could have introduced bias or highlighted differences in practice among surgeons and may have accounted for the overall low proportion of patients that were ultimately selected for fast tracking. While no formal fast tracking protocol exists at our centre, the ability to successfully decide who would be an optimal candidate for fast tracking and who would not is a derivative of years of experience that have afforded the attending surgeons and the nurses looking after these patients, both in the ICU and in the step-down unit, the judgement needed to determine which patients may be successfully fast tracked. Secondly, data are not available on the number of patients who were eligible for fast tracking but who either failed to fast track because they were not clinically ready to be transferred or because they were unable to transfer for non-clinical reasons (lack of bed availability, practitioner unwillingness). In the context of this study, these patients were considered “controls” and were combined with those in whom fast tracking was never considered.

## Conclusion

In conclusion, our study demonstrated that fast tracking was effective in reducing ICU resource utilization while maintaining similar rates of post-operative adverse events. At a time when large teaching hospital ICUs across Canada are operating at or above capacity for significant periods of time [25] and when crises such as the COVID-19 pandemic place even greater strains on an already burdened system [26], the findings from this study may provide the evidence necessary to encourage practitioners to adopt or increase the utilization fast tracking in their respective centres.

## Supplementary Information

**Additional file 1: Table S1.** Baseline characteristics of patients, matched. **Table S2.** Intraoperative characteristics, matched. **Table S3.** In-hospital outcomes, matched.

## Data Availability

All data generated or analysed during this study are included in this published article.

## Abbreviations

BiPAP: Bilevel positive airway pressure
CABG: Coronary artery bypass graft
COPD: Chronic obstructive pulmonary disease
CPB: Cardiopulmonary bypass
CVA: Cerebrovascular accident
CVD: Cardiovascular disease
EMH: Extramural home care
EF: Ejection fraction
ICU: Intensive care unit
LOS: Length of stay
MI: Myocardial infraction
NYHA: New York Heart Association
PVD: Peripheral vascular disease
TIA: Transient ischemic attack
UTI: Urinary tract infection
XC: Cross clamp
