# Supplementary material for: Fast tracking in cardiac surgery: is it safe?
Source: J Cardiothorac Surg. 2022 Apr 6;17:69. doi: 10.1186/s13019-022-01815-9 (PMC8983083; doi:10.1186/s13019-022-01815-9)
Supplement: Supplementary file 1 — Additional file 1: Table S1. Baseline characteristics of patients, matched. Table S2. Intraoperative characteristics, matched. Table S3. In-hospital outcomes, matched. [file 13019_2022_1815_MOESM1_ESM.docx]

Supplementary Table 1: Baseline Characteristics of Patients, Matched

| Characteristics, n (%) | Fast Track (n=245) | Control (n=245) | p-value |
| --- | --- | --- | --- |
| Age, years, mean ± SD | 63 ± 8 | 63 ± 8 | 0.48 |
| Age ≥ 70 years | 58 (23.7) | 52 (21.2) | 0.59 |
| Female sex | 41 (16.7) | 49 (20.0) | 0.41 |
| Smoking history | 165 (67.3) | 164 (66.9) | 1.00 |
| Hypertension | 174 (71.0) | 172 (70.2) | 0.92 |
| Dyslipidemia | 189 (77.1) | 183 (74.7) | 0.60 |
| Diabetes | 88 (35.9) | 100 (40.8) | 0.31 |
| CVD | 15 (6.1) | 15 (6.1) | 1.00 |
| PVD | 19 (7.8) | 18 (7.3) | 1.00 |
| Recent MI ≤ 21 days | 84 (34.3) | 93 (38.0) | 0.45 |
| Renal failure | 2 (0.8) | 1 (0.4) | 1.00 |
| Unstable angina | 137 (55.9) | 141 (57.6) | 0.78 |
| Atrial fibrillation | 12 (4.9) | 13 (5.3) | 1.00 |
| COPD | 11 (4.5) | 11 (4.5) | 1.00 |
| NYHA class 4 | 167 (68.2) | 176 (71.8) | 0.43 |
| EF < 40% | 10 (4.1) | 7 (2.9) | 0.62 |
| Urgent status | 161 (65.7) | 169 (69.0) | 0.50 |

COPD, chronic obstructive pulmonary disease; CVD, cardiovascular disease; EF, ejection fraction; MI, myocardial infraction; NYHA, New York Heart Association; PVD, peripheral vascular disease

Supplementary Table 2: Intraoperative Characteristics, Matched

| Characteristics, n (%) | Fast Track  n=245 | Control  n=245 | p-value |
| --- | --- | --- | --- |
| Procedure | | | |
| CABG | 220 (89.8) | 207 (84.5) | 0.21 |
| Valve | 21 (8.6) | 31 (12.7) |  |
| CABG + valve | 4 (1.6) | 7 (2.9) |  |
| CPB time, mdn (IQR) | 79 (64-95) | 81 (66-97) | 0.47 |
| XC time, mdn (IQR) | 60 (46-73) | 57 (46-75) | 0.97 |
| Inotropes | 29 (11.8) | 32 (13.1) | 0.78 |

CABG, coronary artery bypass graft; CPB, cardiopulmonary bypass; XC- cross clamp

Supplementary Table 3: In-hospital outcomes, matched

| Characteristics, n (%) | Fast Track  n=245 | Control  n=245 | p-value |
| --- | --- | --- | --- |
| *Adverse post-operative outcome* | | | |
| Mortality | 0 (0.0) | 1 (0.4) | 1.00 |
| Reop for bleeding | 0 (0.0) | 2 (0.8) | 0.50 |
| Wound infection | 13 (5.3) | 5 (2.0) | 0.09 |
| UTI | 1 (0.4) | 3 (1.2) | 0.62 |
| Sepsis | 0 (0.0) | 1 (0.4) | 1.00 |
| Atrial fibrillation | 70 (28.6) | 80 (32.7) | 0.38 |
| Renal failure | 6 (2.4) | 4 (1.6) | 0.75 |
| CVA | 1 (0.4) | 1 (0.4) | 1.00 |
| TIA | 0 (0.0) | 0 (0.0) | - |
| *ICU resource utilization* | | | |
| Initial vent time, h | 4.3 (3.3-5.2) | 5.3 (4.1-7.0) | < 0.0001 |
| Reintubation | 0 (0.0) | 1 (0.4) | 1.00 |
| BiPAP | 0 (0.0) | 0 (0.0) | - |
| Initial ICU time, h | 7.8 (6.5-9.4) | 20.6 (17.4-22.3) | < 0.0001 |
| Prolonged ventilation > 24 h | 0 (0.0) | 0 (0.0) | 1.00 |
| Readmission to ICU | 0 (0.0) | 1 (0.4) | 1.00 |
| *Hospital resource utilization* | | | |
| Postop LOS, days, median (IQR) | 5 (4-6) | 5 (4-6) | 0.02 |
| Postop LOS > 5 days | 72 (29.4) | 93 (38.0) | 0.06 |
| *Discharge disposition* | | | |
| Expired | 0 (0.0) | 1 (0.4) | 0.10 |
| Home | 212 (86.5) | 201 (82.0) |  |
| Home EMH | 12 (4.9) | 11 (4.5) |  |
| Other service | 2 (0.8) | 0 (0.0) |  |
| Other institution | 19 (7.8) | 33 (13.5) |  |

BiPAP, bilevel Positive Airway Pressure; CVA, cerebrovascular accident; EMH, extramural home care; TIA, transient ischemic attack; UTI, urinary tract infection
